# The Dopaminergic System in Autoimmune Diseases

**DOI:** 10.3389/fimmu.2014.00117

**Published:** 2014-03-21

**Authors:** Rodrigo Pacheco, Francisco Contreras, Moncef Zouali

**Affiliations:** ^1^Laboratory of Neuroimmunology, Fundación Ciencia & Vida, Santiago, Chile; ^2^Programa de Biomedicina, Universidad San Sebastián, Santiago, Chile; ^3^Universidad Andrés Bello, Facultad de Ciencias Biológicas, Santiago, Chile; ^4^INSERM UMR 1132, Paris, France; ^5^University Paris Diderot, Paris, France

**Keywords:** dendritic cell, regulatory T cell, Th17, Crohn’s disease, multiple sclerosis, ulcerative colitis, systemic lupus erythematosus, rheumatoid arthritis

## Abstract

Bidirectional interactions between the immune and the nervous systems are of considerable interest both for deciphering their functioning and for designing novel therapeutic strategies. The past decade has brought a burst of insights into the molecular mechanisms involved in neuroimmune communications mediated by dopamine. Studies of dendritic cells (DCs) revealed that they express the whole machinery to synthesize and store dopamine, which may act in an autocrine manner to stimulate dopamine receptors (DARs). Depending on specific DARs stimulated on DCs and T cells, dopamine may differentially favor CD4^+^ T cell differentiation into Th1 or Th17 inflammatory cells. Regulatory T cells can also release high amounts of dopamine that acts in an autocrine DAR-mediated manner to inhibit their suppressive activity. These dopaminergic regulations could represent a driving force during autoimmunity. Indeed, dopamine levels are altered in the brain of mouse models of multiple sclerosis (MS) and lupus, and in inflamed tissues of patients with inflammatory bowel diseases or rheumatoid arthritis (RA). The distorted expression of DARs in peripheral lymphocytes of lupus and MS patients also supports the importance of dopaminergic regulations in autoimmunity. Moreover, dopamine analogs had beneficial therapeutic effects in animal models, and in patients with lupus or RA. We propose models that may underlie key roles of dopamine and its receptors in autoimmune diseases.

Regulation of the immune system’s activities by other organs occurs primarily through interactions with receptors expressed on immune cells. Over the last years, studies of the neuroendocrine and immune systems have indicated that neuropeptides, neurotransmitters, hormones, and cytokines, as well as their respective receptors, can be used as common mediators in a neuro-endocrine–immune network, allowing the body to mount proper responses to changes of the internal environment and external insults (1). Interactions between the nervous and immune systems occur through the hypothalamic–pituitary axis and through sympathetic/parasympathetic innervations of primary and secondary lymphoid organs. Remarkably, several neurotransmitter receptors, including cholinergic receptors are expressed by human peripheral blood lymphocytes (2). Among the critical transmitters involved in neuroimmunolgical connections are catecholamines produced by sympatho-adrenergic termini, which can release both dopamine and/or norepinephrine.

Several studies have shown that immune system cells can be regulated by dopamine acting on immune cells expressing dopamine receptors (DARs) present on the surface of T cells, dendritic cells (DCs), B cells, NK cells, neutrophils, eosinophils, and monocytes (3, 4). The presence of these receptors on immune cells suggests that dopamine plays a physiological role in the regulation of the immune response and that its deregulation could be involved in the development of autoimmunity and, even, cancer (4, 5). Furthermore, it implies that different physiological or pathological processes in the nervous system could be involved in the regulation of immune response. On the other hand, several studies show that certain immune cells can synthesize and store dopamine in intracellular vesicles and, upon specific stimuli, release it (6), suggesting that dopamine operates as a bidirectional mediator between nervous cells and immune cells. Here, we discuss the involvement of the dopaminergic system in the pathogenesis of autoimmune diseases.

## Neuroimmune Interactions in Central Nervous System Homeostasis

Immune cells can be exposed to dopamine from several sources. Plasma dopamine levels, which fluctuate depending on autonomic nervous system activity and which are altered in various diseases, are probably the primary source of dopamine available to immune cells present in the blood stream (6). In this regard, chromaffin cells in suprarenal glands may be the main source of plasma dopamine when stimulated by the autonomic nervous system (7). Another peripheral source of dopamine is the gut (8). Gastrointestinal (GI) dopamine is produced by aromatic amino acid decarboxylase (AADC) expressed in epithelial cells in the gut lumen and luminal l-DOPA (9). There are three additional sources of dopamine: immune cells synthesizing and releasing dopamine, the peripheral nervous system (PNS), and the central nervous system (CNS).

The CNS parenchyma and cerebrospinal fluid (CSF) can be important sources of dopamine for immune cells. Entrance of immune system cells in the CNS parenchyma is restricted primarily by the brain–blood barrier (BBB) that surrounds parenchymal venules. The BBB is comprised of a first layer of endothelial cells interconnected by tight junctions, which are surrounded by a basement membrane, and an outer layer constituted by astroglial endfeet (10). Under physiological conditions, no immune cells are found in the CNS parenchyma. However, some immune cells may infiltrate into the CSF, which is produced by choroid plexus epithelia and flows into the subarachnoid space. Importantly, the CSF drains into cervical lymph nodes, enabling peripheral immune cells to recognize and respond to CNS antigens in the absence or presence of inflammation (11, 12). Accordingly, the subarachnoid space and choroid plexus of healthy mice contain substantial numbers of T cells and are heavily populated by myeloid cells, including DCs (13). Importantly, under normal conditions, immune cells found in the subarachnoid space are involved in surveillance of the CNS. When activated by environmental/psychological stimuli, they can regulate nervous system processes, such as memory consolidation, hippocampal long-term potentiation (LTP), neurogenesis, and psychological stress (14). In this regard, recent studies have demonstrated that spatial memory and learning in healthy animals are regulated by the presence of CD4^+^ T cells in the subarachnoid space (15, 16). Whereas recombination-activating gene 2 (RAG2) knockout mice (RAG2KO), devoid of mature T cells and B cells, show impaired performance in Morris-water maze (MWM) tasks, μMT mice (deficient in B cells) display normal learning and spatial memory, indicating that T cells, but not B cells, are required to acquire memory (16). Moreover, class II MHC-deficient mice, which are deficient in CD4^+^ T cells, but whose CD8^+^ T cell compartment remains functionally intact, display significant impairment in MWM tasks (16). Along the same lines, RAG1KO mice, which are, like RAG2KO mice, deficient in T and B cells, improve their learning capability in MWM tasks upon transfer of wild-type (WT) CD4^+^ T cells (16), further indicating the important contribution of the CD4^+^ T cell compartment to the acquisition of spatial memory. Importantly, pharmacological interventions of WT mice with drugs that avoid entrance of T cells into the meningeal compartment, such as FTY720 or anti-VLA4 antibody, impair acquisition of spatial memory (15). In particular, IL-4-producing T cells accumulate in the subarachnoid space during cognitive tasks. In turn, IL-4 produced by Th2 cells stimulates hippocampal astrocytes to produce and release brain-derived-neurotrophic-factor (BDNF) and probably other as yet unidentified factors, which act subsequently on hippocampal neurons, favoring spatial memory and learning (15). Such studies demonstrated an impaired learning in mice lacking CD4^+^ T cells or in mice bearing IL-4-deficient CD4^+^ T cells. In both cases, normal learning is recovered when WT CD4^+^ T cells were transferred, thus linking immune activity to steady-state cognitive functions. More recently, it has been shown that transfer of monoclonal transgenic CD4^+^ T cells bearing T cell receptors (TCRs) specific for CNS antigens into RAG1KO mice, but not CD4^+^ T cells bearing an ovalbumin-specific TCR, leads to improvement of the animal’s capacity to acquire normal learning and spatial memory (16, 17). Similar to the contribution of CD4^+^ T cells to acquisition of spatial memory, there is growing evidence indicating that immunity to self-antigens contributes to psychological stress resiliense (18). Thus, the evidence suggests that T cells recognizing self-antigens from the CNS are necessary to CNS homeostasis. The importance of T cells in cognitive functions is also supported by observations made during aging, HIV infection, and chemotherapy, conditions associated with decreased or impaired T cell functions, and cognitive impairments (14, 17). Potential dopaminergic regulation of T cells and myeloid cells in the meningeal compartment remains to be explored.

## Immune Reactions during Neuroinflammation and Neurodegeneration

As noted above, neuroimmune interactions in the CNS take place in steady-state conditions. Additionally, the presence of infiltrating immune cells in the CNS parenchyma has been detected in most neurodegenerative diseases studied (19). In a pathological scenario involving the CNS, such as neurodegeneration or imbalance of glial homeostasis, initial neuroinflammatory processes induce brain endothelial cells to express a specialized pattern of adhesion molecules on the cell surface. Adhesion molecules induced by inflammatory processes subsequently allow activated T cells to adhere to vessel walls and to be recruited into the CNS parenchyma. Presumably, T cells that infiltrate the CNS have previously been activated in the periphery, in cervical lymph nodes (12, 16). Once in the CNS parenchyma, infiltrating T cells can contribute to regulate the neurodegenerative process by the secretion of different cytokines and the recruitment of innate immune cells. Additionally, molecules derived from immune cells can act over glial cells, modulating microglia phenotype and function, including M1-like microglia, which mediates neurotoxicity, and M2-like microglia, which promotes neuroprotection (20). In this regard, recent studies have shown that peripheral T cells infiltrate into the brain parenchyma at the site of neuronal injury in Parkinson’s disease, where they play a fundamental role in neurodegeneration (21–23). This T cell-mediated immune response contributes significantly to the destruction of dopaminergic neurons, through a CD4^+^ T cell-dependent cytotoxic mechanism. These studies support the involvement of pathogenic CD4^+^ T cell populations in the acquisition of an M1-like pro-inflammatory phenotype by microglia characterized by the secretion of inflammatory factors, such as TNF-α, IL-1β, and superoxide (20). Importantly, dopamine receptor D3 (DAR3) expressed in CD4^+^ T cells is fundamental for promoting destruction of dopaminergic neurons in the substantia nigra in a mouse model of Parkinson’s disease (22). As a result, DAR3-deficient (DAR3KO) mice are resistant to 1-methyl-4-phenyl-1,2,3,6-tetrahydropyridine (MPTP)-induced Parkinson’s disease. However, when WT CD4^+^ T cells were transferred into DAR3KO mice, they acquired the capability to respond to MPTP-induced neurodegeneration. On the other hand, RAG1KO mice, which are resistant to MPTP-induced Parkinson’s disease, acquire the capacity to respond to MPTP-induced neurodegeneration when WT, but not DAR3KO, CD4^+^ T cells were transferred (22). Furthermore, CD4^+^ T cells that infiltrate in the substantia nigra during MPTP-induced Parkinson’s disease produced high levels of IFN-γ and TNF-α, two cytokines that act synergistically in microglia to promote the inflammatory M1-like phenotype (24). Thus, these findings point toward an important role of CNS-derived dopamine in the regulation of T cell-mediated immunity during neuroinflammation. Conversely, other T cell subsets, i.e., regulatory T cells (Tregs) and Th2 cells, could contribute to microglial acquisition of an M2-like anti-inflammatory phenotype, releasing neurotrophic factors, such as IGF-1, and promoting neuronal protection (20, 23). In contrast to the role of CNS-infiltrating T cells in Parkinson’s disease, studies carried out in amyotrophic lateral sclerosis (ALS) have shown that absence of T cells accelerates motoneuron disease while adoptive transfer of T cells ameliorates disease severity (19). These results could be explained by the contribution of T cells to the acquisition of M2-like phenotype by microglia. Consistently, both Treg numbers and FoxP3 protein expression were reduced in rapidly progressing ALS patients and inversely correlated with progression (25). Interestingly, expression of two Th2 molecular markers, GATA3 and IL-4, was also decreased in peripheral T cells in rapidly progressing patients and inversely correlated with progression rates (25). Thus, an imbalance of neuroimmune interactions may constitute an important component of the pathogenic mechanisms involved in neurodegenerative disorders regulating the onset and progression, and dopamine may be a key mediator in these neuroimmune interactions, such as demonstrated for Parkinson’s disease (22).

## Sympathetic Nervous System-Mediated Regulation of Immunity

An important peripheral source of dopamine and other neurotransmitters is the innervation of primary and secondary organs by the sympathetic nervous system (SNS). For example, dopaminergic terminals have been detected in thymus, spleen, and lymph nodes where the SNS seems to play a role in regulation of T cell-mediated responses (26). The relevance of SNS-mediated regulation of immunity has been demonstrated by several studies that were mainly carried out in mice that received 6-hydroxydopamine, a neurotoxic drug that selectively ablates noradrenergic and dopaminergic neurons. This latter molecule is captured specifically through dopamine transporters (DAT) or norepinephrine transporters (NET), and, when administered systemically, it cannot cross the BBB. As a result, 6-hydroxydopamine depletes the SNS without affecting the CNS. NE is the main neurotransmitter released by the SNS and, thereby, this is probably the main neurotransmitter responsible for SNS-mediated regulation of immunity, although dopamine has also been involved in SNS-mediated regulation of T cell responses. The evidence points to a dual role of the SNS in T cell responses. First, the SNS inhibits TGF-β production in the spleen and lymph nodes, thereby attenuating generation and function of Tregs. Thus, sympathectomy attenuates disease severity of mice with collagen-induced arthritis (CIA) or experimental autoimmune encephalomyelitis (EAE) as compared with animals bearing intact SNS (27, 28). Second, by stimulating β2-adrenergic receptors, the SNS attenuates immunogenicity of antigen-presenting cells (APCs) in secondary lymphoid organs, decreasing the potency of both Th1 response and CD8^+^ T cell-mediated cytotoxicity. This attenuation seems to alter the immune response during infection with influenza virus (29) or *Listeria monocytogenes* (30). In both cases, sympathectomized mice mount a stronger antiviral or antibacterial response than control mice (29, 30). Thus, the SNS displays a dual role, potentiating autoimmune responses and attenuating antiviral and antibacterial responses. This dual function could be explained by a mechanism in which SNS-mediated attenuation of Th1 responses concomitantly favors Th17 responses. Another mechanism could involve dopamine. Indeed, SNS neurons express the enzyme dopamine-β-hydroxylase (DβH), which catalyzes the synthesis of NE from dopamine, a neurotransmitter normally present in low amounts in mouse SNS neurons. However, in DβH-deficient mice (DβHKO), noradrenergic neurons become exclusively dopaminergic, and the mice develop an attenuated antibacterial response against *L. monocytogenes* (31). Thus, exacerbated dopamine signaling by the SNS results in a decreased Th1 response.

## Immune Cells as a Source of Dopamine

**Figure 1:**
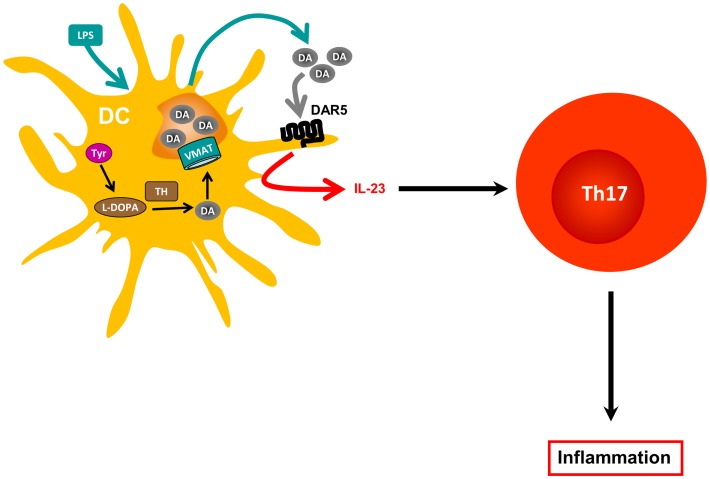
**Dopamine produced by dendritic cells and amplification of inflammation**. Dendritic cells (DCs) express tyrosine hydroxylase (TH), which catalyzes the first step required for dopamine (DA) biosynthesis. However, since these cells do not express dopamine β-hydroxylase, the enzyme required to metabolize DA and to transform it into epinephrine and norepinephrine, they accumulate DA. DCs also express vesicular monoamine transporters 1 and 2 (VMAT) required to store DA in vesicular compartments. In response to antigen presentation or to LPS stimulation, DCs release DA from intracellular stores, which can modulate both DC physiology in an autocrine manner and CD4^+^ T cell responses in a paracrine fashion (not depicted). At certain concentrations, DC-derived DA interacts with DAR5 expressed by DCs, which promotes IL-23 production in response to LPS, and, thereby, enhances Th17 responses.

An increasing number of studies revealed that cells involved in both adaptive and innate immune responses, such as DCs, T cells, B cells, and macrophages are capable of synthesizing neurotransmitters (32). Under specific stimuli, these cells may release neurotransmitters into the extracellular compartment, thus enabling communications with other different cell types. These interactions not only suggest that neurotransmitters can mediate communication between immune cells, but also that these molecules may be involved in a bidirectional cross-talk between the immune and the nervous system. With regard to dopamine, early studies showed that *in vitro* activation of human peripheral blood mononuclear cells (PBMCs) with mitogens induces production of intracellular dopamine and other catecholamines, probably involving both T and B lymphocytes (33, 34). Currently, several studies performed in human and mouse cells indicate that DCs and Tregs constitute dopamine sources. DCs express tyrosine hydroxylase (TH), which catalyzes the first step required for dopamine biosynthesis (Figure 1). However, these cells do not express dopamine-β-hydroxylase, the enzyme required to metabolize dopamine and to transform it into epinephrine and NE (35). In addition, DCs do not express DAT, required to take up dopamine from the extracellular compartment. Thus, DCs synthesize dopamine, but not other catecholamines, and they cannot capture dopamine from the extracellular space. These cells also express enzymes necessary to degrade dopamine in the cytoplasm: monoaminooxidases A and B (MAO-A, MAO-B) and vesicular monoamine transporter 2 (VMAT-2) required to store dopamine in vesicular compartments. In addition, human DCs contain intracellular dopamine, which is released upon antigen presentation to T cells (36). On the other hand, human Tregs constitutively express TH and contain substantial amounts of dopamine and other catecholamines, while effector T cells only contain trace amounts (37). Tregs also express VMAT-1 and VMAT-2, which allows them to accumulate catecholamines in vesicular stores (37). Interestingly, physiologically relevant amounts of dopamine are released by Tregs when stimulated by reserpine, a natural drug used to deplete monoamines (37). Addressing the physiological stimuli evoking release of intracellular catecholamines from lymphocytes, *in vitro* treatment of mitogen-stimulated PBMCs with IFN-β induces a stronger production of catecholamines and the release of these mediators into the culture supernatant (38), thus suggesting that IFN-β is an endogenous stimulus for secretion of catecholamines from lymphocytes. Other immune cells have been described to store dopamine in intracellular compartments. In this regard, stimulation of B cells with mitogens induces up-regulation of TH mRNA expression followed by production of intracellular dopamine and other catecholamines by a PKC-dependent mechanism (34). In addition, it has been shown that intracellular vesicles containing dopamine in B cells can be released by Ca^2+^-dependent mechanisms (39). Similarly, other studies suggest the existence of dopamine-containing vesicles in monocytes/macrophages (39, 40). Neutrophils and mast cells have been also suggested to contain dopamine (39, 41). Further experimental work is necessary to understand the relevance of these immune cells as sources of dopamine in neuroimmune interactions and in leukocyte–leukocyte communications.

## Dopamine Receptor Expression in the Immune System

Dopamine receptors have been found not only in cells of the innate immune response such as DCs, NK cells, macrophages/monocytes, and granulocytes (35), but also in cells of the adaptive immune response, such as B cells, CD8^+^ T cells, and CD4^+^ T cells (36, 42–45). DARs expression has also been described in murine T cells (45, 46). Pharmacological evidence obtained from studies performed with human T cells has suggested that, among the five known DARs (DAR1–DAR5), both type I (DAR1 and DAR5) and type II (DAR2, DAR3, and DAR4) receptors contribute to the regulation of T cell functions. It has been suggested that stimulation of type I DARs expressed on human naive CD4^+^ T cells potentiates the production of Th2 cytokines (36). Other investigators have suggested that stimulation of type I DARs on human Tregs can decrease IL-10 and TGF-β production and their suppressive activity (37), and that DAR4 stimulation on human T cells promotes quiescence (44). On the other hand, there is evidence that stimulation of DAR2 and DAR3 in normal human resting T cells favors production of IL-10 and TNF-α, respectively (42), and that stimulation of DAR3 in resting T cells favors activation of β1-integrins and adhesion to fibronectin, two critical events required for cell migration (45). Importantly, DAR3 stimulation in human activated CD4^+^ T cells decreases IL-4 and IL-10 synthesis and potentiates IFN-γ production, a key cytokine for Th1 cells; and pharmacologic DAR3 stimulation in human T cells potentiates expression of surface activation markers (47). In contrast, other studies indicated that dopamine, at concentrations that selectively stimulate DAR3, inhibits human T cell proliferation (43, 48). In addition to the different stimulatory effects of DARs in T cell physiology, it is important to consider that each DAR displays different affinities for dopamine: DAR3 > DAR5 > DAR4 > DAR2 > DAR1 [Ki (nM) = 27, 228, 450, 1705, 2340, respectively] (49–51). Thus, low levels of dopamine, e.g., 50 nM, would stimulate mainly DAR3 in T cells, favoring Th1-like responses and T cell migration, whereas moderate dopamine levels, e.g., 300 nM, would stimulate DAR5 as well, inhibiting T cell function. It is likely that, by stimulating multiple DARs, higher dopamine levels promote complex effects in T cells, probably inhibiting T cell-mediated immunity (4).

**Figure 2:**
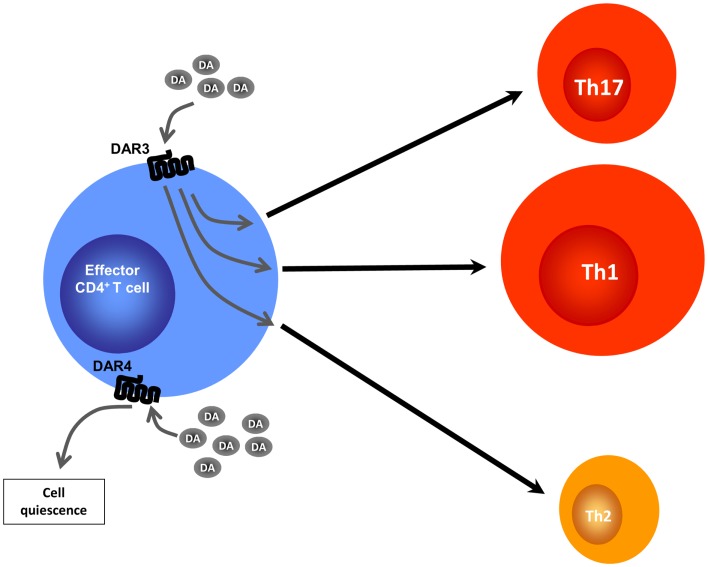
**Altered CD4^+^ T cell programing by different dopamine concentrations**. Effector CD4^+^ T cells lack the ability to synthesize DA, but may be exposed to DA produced by DCs, Tregs, or by other sources (not depicted). At intermediate DA concentrations, stimulation of DAR4 expressed by effector CD4^+^ T cells leads to cell quiescence by inducing expression of KLF2, a transcription factor that regulates T cell quiescence. At lower DA concentrations, stimulation of DAR3 expressed on DCs results, by means of an undefined mechanism, in heightened Th1 responses along with a reduction in Th17 immunity and a reduction of Th2-related cytokines.

Regarding DCs, DAR1 and DAR5 are expressed at higher levels on the cell surface, whereas DAR3 and DAR2 are poorly represented on DCs (35). It has recently been demonstrated that stimulation of DAR5 on DCs strongly potentiates production of IL-23, a regulatory cytokine that favors polarization of naive CD4^+^ T cells toward the inflammatory Th17 phenotype (Figure 2). Specifically, stimulation of DAR5 on DCs potentiates Th17 responses *in vitro* and *in vivo* (35). Other pharmacological evidence indicates that selective stimulation of DAR2/DAR3 or selective inhibition of DAR1/DAR5 on DCs favors polarization of CD4^+^ T cell responses toward Th1, but impairs the Th17 fate (52). Thus, depending on the concentration of dopamine, the specific DARs expressed and the type of immune cell bearing DARs, this neurotransmitter may induce different effects in the immune response.

Emerging evidence has shown association of some autoimmune disorders with abnormal dopamine levels and deregulation of dopaminergic components expressed in immune cells. For example, dopamine levels are altered in the brain of mouse models of multiple sclerosis (MS) and systemic lupus erythematosus (SLE), and in inflamed tissues of patients with inflammatory bowel diseases (IBDs) or rheumatoid arthritis (RA). The distorted expression of DARs in peripheral lymphocytes of SLE and MS patients supports the importance of dopaminergic regulations in autoimmunity. Here, we critically discuss the role of dopaminergic regulation of the immune response and its implications with regard to autoimmune disorders.

## Cell Subsets Involved in Multiple Sclerosis Pathogenesis

Multiple sclerosis is a chronic demyelinating disease resulting from an autoimmune response against constituents of the CNS. With approximately 2.4 million individuals affected world wide, the disease is characterized by CD4^+^ T cell-mediated progressive loss of neurological function due to the destruction of axonal myelin sheath in several areas of the brain and spinal cord. Loss of myelin is manifested in clinical symptoms such as paralysis, muscle spasms, optic neuritis, and neuropathic pain (53). Pathological features of MS lesions include BBB permeability, myelin sheath destruction, axonal damage, glial scar formation, and presence of lymphocytes and inflammatory cells infiltrated into the CNS (54). Despite intense investigation, the etiology of MS is still unclear; but genetic and environmental factors have been suggested to be important in disease development.

Several cell types are present in the CSF and microvasculature. An increased number of DCs with an exacerbated state of maturation have been found in the CSF and lesions of patients with MS (55). It is thought that, in healthy individuals, CNS-resident DCs play a role in the induction of peripheral tolerance to CNS-derived self-constituents, including myelin. However, due to the abnormal mature phenotype of DCs and their proximity with infiltrated T cells in the CNS of MS patients, DCs could play an important role in presenting self-antigens and in re-stimulating self-reactive T cells infiltrated into the CNS. In addition, peripheral blood DCs from MS patients produce increased amounts of IL-6, IFN-γ, and TNF-α (56). Importantly, these DCs are capable of promoting differentiation of CD4^+^ T cells toward the inflammatory Th17 phenotype (57). These observations support the notion that abnormal function of DCs from MS patients can promote an inappropriate functional phenotype in self-reactive T cells, contributing to the development of autoimmunity. In addition to CD4^+^ T cells, CD8^+^ T cells have also been described as an important cell subset in the disease (58). In fact, the frequency of CD8^+^ T cells is greater than that of CD4^+^ T cells in inflamed MS plaques, and CD8^+^ T cells show oligoclonal expansion in plaques, CSF, and blood, suggesting an important role of these cells in MS. In this regard, a group of studies performed in mice and in humanized mice showed participation of CD8^+^ T cells in EAE (58). B cells are also important by promoting the autoimmune response involved in MS; and it has been suggested that IL-6-producing B cells are the pathogenic cell subset that promotes functions of self-reactive T cells (59). Importantly, treatment with rituximab, an anti-CD20 antibody that alleviates symptoms of MS patients (60), induces preferential depletion of IL-6-producing B cells (59).

## Dopaminergic Regulation in Multiple Sclerosis

Experimental autoimmune encephalomyelitis is a widely used mouse model of MS. It can be induced by injection of a peptide derived from the myelin-oligodendrocyte glycoprotein (MOG), MOG_35–55_ (pMOG), emulsified with adjuvant (35). Administration of myelin-derived antigens in an immunogenic context promotes activation of self-reactive T cells specific for myelin antigens, which mediate myelin destruction characterized by focal areas of demyelination along the CNS, with axonal loss that results in ascending paralysis, affecting first the tail and then the hind limbs. During EAE onset and at the peak of disease manifestations, there is an important increase of dopamine levels in the striatum nucleus (61). Interestingly, levels of IL-1β and TNF-α mRNAs increase in the striatum nucleus with the same kinetics followed by dopamine (61). On the other hand, there is decreased expression of DAR5 in PBMCs obtained from untreated MS patients when compared with PBMCs obtained from healthy donors (62). Furthermore, when patients are treated with IFN-β, there is a progressive increase of the expression of DAR5 in PBMCs during the treatment period (63). It is possible that decreased expression of DAR5 on PBMCs could be a pathogenic factor favoring disease manifestations. Alternatively, DAR5 expressed on these cells could represent a detrimental factor in MS; and, somehow, a compensatory down-regulation of this receptor could be promoted in MS patients. Moreover, altered production of catecholamines by PBMCs, which is modulated through type I DARs (34), seems also to occur in PBMCs from MS patients (33). Importantly, IFN-β, which has been shown to be therapeutic for the treatment of MS, promotes a progressive increase of TH expression with a consequent increase of catecholamines production in PBMCs (63). It is noteworthy that IFN-β is capable to induce release of catecholamines from PBMCs *in vitro* (38); however, the physiological relevance of this IFN-β function remains unclear. These results suggest a relationship between expression of components of the dopaminergic system in immune cells and MS development (see Table 1).

**Table 1:** **Observations linking the dopaminergic system with the development and progression of autoimmune disease**.

Disease	Experimental mouse models	Reference	Patients	Reference
Multiple sclerosis	Increased dopamine levels in the striatum nucleus during EAE peak	(61)	Decreased expression of DAR5 in PBMCs obtained from untreated MS patients compared with healthy donors	(62)
	Administration of the DAR1-like antagonist SCH-23390 prevents the development of EAE	(52)	Increased expression of DAR5 and TH, along with elevated catecholamine content in PBMCs of MS patients treated with IFN-β	(63)
	Stimulation of DAR5 on DCs promotes Th17-driven EAE	(35)	Treatment of MS patients with IFN-β reduces high levels of DAR5 and TH expressed on Tregs and abolishes dopamine-mediated inhibition of suppressive activity of Tregs	(66)
Inflammatory bowel diseases	TNBS-induced colitis is associated with reduced tissue levels of dopamine	(96)	Inflamed gut mucosa from CD and UC patients shows a marked reduction of dopamine content	(95)
	6-Hydroxydopamine-induced sympathectomy increases gut inflammation in chronically DSS-treated and IL-10-deficient mice	(97)	CD patients have reduced numbers of sympathetic fibers interacting with the intestinal wall	(97)
	Dopamine acts via DAR2 to suppress both increased motility and ulcer development induced by chemical insult	(102)	A genetic polymorphism of DAR2 gene, which results in reduced receptor expression, has been reported as a risk factor to develop refractory CD	(103)
Rheumatoid arthritis	Treatment with the selective DAR1-like antagonist SCH-23390 suppresses collagen-induced arthritis severity, probably due to inhibition of macrophage differentiation into osteoclasts	(108)	Dopamine is significantly increased in the synovial tissue of RA patients	(107)
	Selective DAR2-like receptor antagonist haloperidol significantly exacerbated cartilage destruction, whereas DAR1-like receptor antagonist SCH-23390 strongly suppresses RA development	(107)	Administration of bromocriptine, a DAR2/3 agonist, suppresses immune parameters and reduces RA disease activity	(112)
	Adoptive transfer of TH^+^ cells generated from mesenchymal stem cells reduces collagen-induced arthritis severity	(111)	Treatment of active RA with cabergoline, a DAR2/3 agonist, significantly reduces disease activity	(113)
			Dopamine-producing TH^+^ leukocytes with anti-inflammatory properties are found in synovial tissue of RA patients, but not in healthy controls	(110)
Systemic lupus erythematosus	Lupus-prone MRL-lpr mice have impaired coordination and neurological deficits, with imbalanced dopamine function and neurodegeneration in dopamine-rich brain regions	(120)	Autoantibodies targeting dopaminergic neurons are associated with rapidly progressing Parkinsonian symptomatology in a SLE patient	(119)
	Brains of MRL-lpr mice show elevated levels of dopamine and increased sensitivity to the DAR2/3 receptor agonist quinpirole, suggesting a neurotoxic role for dopamine	(122)	SLE patients show reduced expression of DAR2 and increased DAR4 levels on PBMC-derived T cells compared to healthy individuals	(123)
	Chronic administration of the selective DAR2/3 agonist quinpirole induces self-injurious behavior in lupic mice	(121)	Treatment with bromocriptine, a DAR2/3 agonist, is beneficial in SLE patients with mild to moderately active disease, leading to decreased serum immunoglobulin and anti-DNA antibody levels	(112)
	Bromocriptine, a DAR2/3 agonist, slows the course of SLE in (NZB × NZW) F1 mice and is effective in treating established disease in this model	(125)		
	Combined treatment with estrogen and bromocriptine, a DAR2/3 agonist, prevents development of a lupus-like syndrome in BALB/c mice expressing a transgenic anti-dsDNA antibody	(126)		

*DC, dendritic cell; CD, Crohn’s disease; EAE, experimental autoimmune encephalomyelitis; DAR, dopamine receptor; MS, multiple sclerosis; TH, tyrosine hydroxylase; UC, ulcerative colitis*.

Of late, it was demonstrated that DCs express the whole machinery to synthesize and store dopamine (35). The dopamine released from DCs subsequently acts in an autocrine manner to stimulate DAR5 expressed on DCs, promoting a potent production of IL-23 by DCs, thus conferring these cells with the ability to favor polarization of naïve CD4^+^ T cells toward the inflammatory Th17 phenotype (35). Since IL-23 can also stimulate γδT cell functions (64) and GM-CSF-producing CD4^+^ T cells (65), two cell subpopulations involved in EAE pathogenesis, the DAR5 expressed on DCs could play a role in EAE by regulating the activity of various target T cell populations. This autocrine loop mechanism mediated by dopamine and stimulation of DAR5 on DCs could represent a driving force in the autoimmune response during EAE development. Supporting this notion, DAR5KO mice manifest EAE with significantly lower severity than WT mice (35). In addition, prophylactic transfer of DAR5KO DCs loaded with pMOG into WT recipient mice significantly reduces EAE severity when compared with WT recipient mice transferred with pMOG-loaded WT DCs (35). In the same direction, another study has shown that systemic administration of a selective antagonist for type I DARs decreased significantly EAE severity (52). Furthermore, attenuated disease severity correlates with decreased numbers of Th17 and IFN-γ^+^ IL-17^+^ CD4^+^ T cells infiltrated into the CNS of WT mice transferred with DAR5KO DCs when compared with those transferred with WT DCs. Conversely, there are no differences in the frequencies of Tregs or Th1 cells infiltrated into the CNS when compared to WT mice transferred with DAR5KO DCs versus those transferred with WT DCs. However, the participation of γδT cells, GM-CSF-producing CD4^+^ T cell, and CD8^+^ Tregs has not been explored.

**Figure 3:**
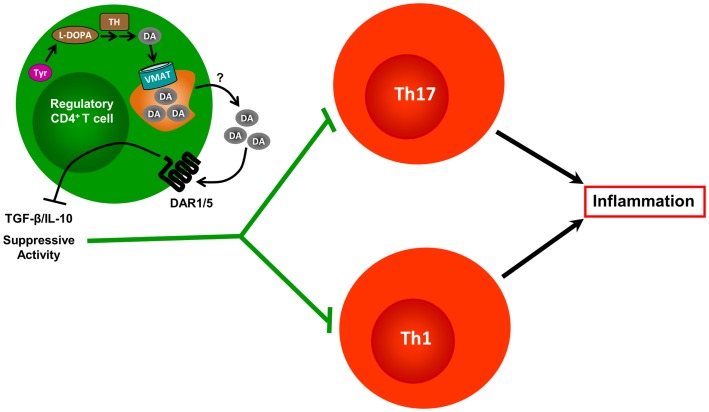
**Contrasted effects of dopamine produced by regulatory T cells**. Tregs constitutively express TH and contain substantial amounts of DA. They also express VMAT-1 and VMAT-2, which allows them to accumulate DA in vesicular stores. In response to yet unknown physiological stimuli, Tregs release DA, which can interact with DARs expressed on the Treg cell surface, but also with DARs present on DCs and effector CD4^+^ T cells (not depicted). Treg-derived DA interaction with DAR1/5 expressed by Tregs, reduces the expression of IL-10 and TGF-β, and weakens the Treg’s suppressive activity exerted over effector CD4^+^ T cells.

Regarding dopaminergic regulation of T cells, the physiologic stimulus that induces dopamine release from Tregs remains unknown. Yet, when treated with reserpine, a drug used to deplete dopamine from dopaminergic cells, Tregs release high amounts of dopamine, which acts in an autocrine manner to inhibit their suppressive activity. Pharmacological evidence suggests that this inhibition of Treg suppressive activity is mediated through type I DARs, including DAR1 and/or DAR5 (37). Accordingly, a recent study that analyzed the suppressive function and expression of the dopaminergic machinery in Tregs obtained from MS patients or from healthy controls found that DAR5, as well as TH, were up-regulated in Tregs from untreated MS patients when compared with those from healthy controls; however, both TH and DAR5 were down-regulated when Tregs were obtained from IFN-β-treated MS patients (66). Whereas the suppressive function was partially or completely inhibited by dopamine when Tregs were obtained from healthy controls or untreated MS patients, respectively, dopamine-mediated inhibition of Treg function was abolished when Tregs were obtained from IFN-β-treated MS patients (66). Interestingly, DAR3 expression in Tregs was unaltered in untreated MS patients, but significantly decreased in IFN-β-treated MS patients when compared with healthy controls (66). Importantly, inhibition of Treg function could be achieved by 1 μM dopamine (66). Considering the affinity of dopamine for DARs expressed on human Tregs (DAR1, DAR3, and DAR5) and the pharmacological evidence obtained using dopamine analogs in these cells (37), the results suggest that the inhibitory effect exerted on Treg suppressive functions is mediated by DAR5, rather than DAR1. Thus, these results support the notion that DAR5 stimulation could be involved in inhibition of Treg suppressive activity (Figure 3). On the other hand, because DAR5 expression is altered in MS patients, these findings also suggest the involvement of DAR5 expressed on Tregs in the physiopathology of MS (see Table 1). Since CD8^+^ T cells and B cells are also involved in MS pathogenesis, and because DARs are expressed in these cell populations, further efforts are necessary to understand the role of dopaminergic regulation of these cells in MS. Moreover, further experimental work is necessary to provide an integrated view of the role of dopaminergic regulation in the autoimmune response during MS. Further understanding of the precise mechanisms involving dopaminergic-mediated regulation of the immune system and their alterations in MS could lead to development of drugs useful for MS treatment.

## Mucosal Immune System in Inflammatory Bowel Diseases

Inflammatory bowel diseases form a group of chronic remittent inflammatory affections of the GI tract, among which Crohn’s disease (CD) and ulcerative colitis (UC) are the most common. The overall IBD prevalence approximates 500–900 cases per 100,000 individuals, and has shown a marked increase during the last decades, mainly in westernized nations (67, 68). In terms of clinical manifestations, both CD and UC share several symptoms such as abdominal pain, anemia, weight loss, and diarrhea, but they also differ in a number of pathological features. Lesions in CD may occur anywhere from the mouth to the anus, with a preference for the ileum, while UC patients typically present lesions restricted to the colon. UC involves mostly mucosal inflammation, while CD is characterized by discontinuous transmural inflammation. In addition, UC may evolve to toxic mega-colon or predispose to colorectal cancer, while CD may progress into perianal fistulas, abscesses, and strictures, which may lead to intestinal obstructions (69). Evidence from IBD mouse models has associated different CD4^+^ T helper lineages as the main drivers of inflammation, linking CD development to Th1/Th17 responses, and UC to Th2 responses (70). More recently, however, both gene and protein expression studies have confirmed that inflamed samples from both UC and CD patients are dominated by Th1/Th17 markers with little or no signs of Th2 contribution, suggesting that established inflammation in IBD is mainly driven by Th1/Th17 cell populations (71, 72). Treatment of these conditions relies on classical anti-inflammatory and immunosuppressive drugs such as mesalamine compounds, corticosteroids, and azathioprine. However, these agents are known to vary in their ability to control the symptoms and to be toxic. Novel therapies rely on neutralizing antibodies raised against inflammatory cytokines such as TNF-α and the p40 subunit of IL-12 and IL-23, which are important in the innate response that polarizes adaptive immunity and perpetuates inflammation (69, 73).

Current understanding of IBD pathogenesis indicates that these disorders are driven by an excessive immune response against normal constituents of the mucosal microbiota in genetically predisposed individuals. Specifically, intestinal microbiota is able to modulate the mucosal immune response and seems to play a key role in IBD pathology (74). Several observations suggest that inflammation *per se* may cause dysbiosis, and severe inflammation might be associated with profound changes in the intestinal microflora, acting as a perpetuating factor in IBD by increasing the contribution of commensals with pathogenic tendencies among the community (75). Additionally, the epithelial barrier plays a crucial role in maintaining mucosal homeostasis as it orchestrates the communication between the intestinal mucosa and luminal contents, and as it establishes an appropriate substrate for commensal microbiota. These various players enable the mucosal immune system to continuously sample luminal contents and to be able to decide between tolerating commensal microflora and dietary antigens, or activating and responding to invading pathogens. Through several mechanisms, sampled antigens are transported to APCs, like DCs and macrophages. Several studies have regarded DCs and macrophages as inducers of intestinal inflammation (76, 77). In contrast to DCs and macrophages, NK cells attenuate intestinal inflammation induced by transfer of IL-10-deficient CD4^+^ T cells into immunodeficient hosts by a mechanism dependent on perforin (78).

Activation of innate immunity leads to recruitment and activation of several components of the adaptive immune response, which mediate damage of intestinal tissues in IBDs and their mouse models. Th1 cells were initially credited as inflammation inducers in experimental colitis. Further studies drew attention to the involvement of Th17 cells in IBDs. Thus, IL-17 expression is increased in both CD and UC, with a higher frequency of IL-17-producing CD4^+^ T cells in colonic mucosa of CD patients (79, 80). In addition, Tregs seem to play a crucial role in maintaining intestinal homeostasis. These cells can suppress inflammation induced by CD4^+^ CD45RB^hi^ T cells in a T cell transfer model of colitis (81); and one of the main suppressive mechanisms relies on IL-10 secretion by these cells. Thus, specific IL-10 deletion of Foxp3^+^ Tregs results in spontaneous colitis, highlighting the fact that IL-10 produced by Tregs is instrumental in maintaining tolerance particularly at intestinal tissues (82). In humans, CD4^+^CD25^+^Foxp3^+^ Treg cells are increased in the inflamed lamina propria of CD and UC patients compared to uninflamed mucosa and mucosa from healthy controls, and they retain their ability to suppress CD4^+^CD25^−^ T cells, suggesting that Treg function is not altered during the course of intestinal inflammation (83, 84). In accordance with these data, T cells from IBD patients were found to be resistant to suppressive molecules, such as TGF-β, resulting in excessive T cell-mediated inflammation (85). Alterations in additional regulatory populations of T cells have been reported in IBDs, such as CD8^+^ CD28^−^ T cells, which are reduced or absent in the lamina propria of IBD patients. Regulatory B cells have also been associated with these diseases, as they are detected in inflamed sites and can suppress the progression, but not the initiation phase of colitis (86, 87). Finally, it is important to note that NKT cells, which respond to phospho- or glycolipids rapidly produce Th1-, Th2-, and Th17-associated cytokines, major drivers of UC pathology (88).

## Dopamine and Mucosal Immunity in Inflammatory Bowel Diseases

Cells residing in the GI tract may encounter the neuromodulator dopamine, which can be produced from different sources, including the enteric nervous system, the intestinal epithelial layer, and certain immune cells, as described above. The intestine receives extrinsic signals from the CNS by means of sympathetic and parasympathetic nerve fibers belonging to the autonomic nervous system. While sympathetic fibers arise from the corresponding spinal nerves, parasympathetic fibers derive from the vagus nerve. Both vagal and spinal fibers connect within the gut wall at different points, interacting with the “intrinsic” enteric nervous system. The latter is organized in three layers, which include the myenteric plexus, located between the circular and longitudinal muscle layers, the submucosal plexus, localized at the submucosa, and the mucosal plexus, which contains nerve endings in close proximity to immune and epithelial cells. This system includes sensory neurons, interneurons, motor neurons, and enteric glial cells, and secretes more than 30 different neurotransmitters, with most neurons expressing multiple neurotransmitters (89).

Dopamine is detectable in the intestine, but it remains unclear if this neuromodulator comes from extrinsic or intrinsic innervations (90). Several lines of evidence support the view that dopamine is produced by intrinsic enteric neurons, since gastric fibers are immunoreactive with dopamine, and because nerve stimulation of the guinea pig stomach results in dopamine release (91). Furthermore DAT immunoreactivity has been detected in enteric nerves (92). In addition, the specific dopamine-derived metabolite dihydroxyphenylacetic acid (DOPAC) has been found in the mouse intestine, and treatment with 6-hydroxydopamine, an agent that ablates neurons expressing DAT or NET, results in depletion of enteric dopamine (90). Enteric dopamine has also been reported in human myenteric neurons, which are depleted in Parkinson’s disease patients (93). Additional studies have shown that TH, dopamine, and DAT immunoreactivities colocalize in subsets of neurons from mouse intestines, known to be resistant to extrinsic denervation, thus strongly suggesting that enteric dopaminergic neurons are intrinsic (94). On the other hand, dopamine may be synthesized by epithelial cells of the intestinal mucosa, which show high AADC activity and are exposed both to circulating or luminal l-DOPA (9). Interestingly, inflamed mucosa from CD and UC patients shows a marked reduction of dopamine content (95), which may alter the activation or differentiation status of DAR-expressing immune cells, such as inflammatory T cells, Tregs, B cells, macrophages, NK cells, and DCs. Consistently, 2,4,6-trinitrobenzene sulfonic acid (TNBS)-induced colitis has been associated with reduced tissue levels of dopamine and no changes in AADC activity (96). The authors hypothesized that the mechanism behind dopamine reduction under inflammatory conditions might be explained by the reduced uptake of l-DOPA by cultured epithelial cells upon IFN-γ treatment (96). However, the *in vitro* model of intestinal epithelial cells is an over-simplified system that lacks many cellular and molecular mediators. An alternative explanation comes from work showing that CD patients have reduced numbers of sympathetic fibers interacting with the intestinal wall, and that 6-hydroxydopamine-induced sympathectomy increases the inflammation in chronically DSS-treated and IL-10-deficient mice, strongly supporting an anti-inflammatory role of sympathetic nerves in IBDs (97). As previously observed for the cholinergic anti-inflammatory reflex, autonomic circuits may relay signals with the use of different neurotransmitters, and, in that way, a cholinergic pathway, i.e., the vagus nerve, may stimulate NE release into the target tissue, i.e., the splenic nerve (98). This could also be the case in the enteric nervous system, where neurons from the enteric nervous system could respond to vagus nerve stimulation by secreting locally relevant neuromodulators, such as dopamine (99). Thus, the reduced sympathetic innervation observed in CD patients could account for reduced levels of dopamine, also observed in mouse models of colitis.

As mentioned above, development of IBD results from an excessive mucosal immune response in genetically predisposed individuals, who may have alterations in one or various processes that ensure mucosal homeostasis. For example, deregulated intestinal motility may lead to lesion induction, and, eventually, to ulcer development, which may predispose to inflammation. In this context, dopamine or its agonists have been reported to act as protective agents in various rat ulcer models (100, 101). More recently, it was shown that dopamine acts via DAR2 to suppress both increased motility and ulcer development induced by chemical insult (102). These findings suggest that, by controlling intestinal motility, dopamine may reduce the propensity to develop ulcers upon intestinal injury. In line with this notion, a genetic polymorphism of DAR2 gene, which results in a reduced receptor expression, has been reported as a risk factor to develop refractory CD (103). Another study showed participation of dopamine acting via DAR1 in smooth muscle inhibition of acute cold/restraint stress-induced contraction in the distal colon of the rat (104). Moreover, DAR5 exhibits immunoreactivity at apical and basolateral sides of intestinal crypts, where its pharmacological stimulation promotes epithelial K^+^ secretion via a cAMP-dependent mechanism, thus ensuring the correct function of the intestinal mucosa (105).

Regarding the expression of DARs by immune cells, DAR5 expressed on DCs seems to favor the expression of both IL-12 and IL-23 in response to LPS, potentiating CD4^+^ T cell activation (35). Considering the crucial role of IL-12 and IL-23 produced by DCs in intestinal inflammation, it is possible that dopamine acting via DAR5 on DCs may promote Th17 differentiation in the gut and contribute to IBDs. Previous work showed that DAR3-deficient CD4^+^ T cells mount impaired Th1 responses and fail to induce neurodegeneration in a murine Parkinson’s disease model (22). Taking into account the reduction in intestinal dopamine levels [≈140 pg/ml in healthy individuals; ≈45 pg/ml in CD and UC patients (95)] and the fact that DAR3 and DAR5 may be stimulated at low dopamine concentrations, it is tempting to speculate that low dopamine levels present in the inflamed gut may drive an inflammatory phenotype in CD4^+^ T cells, thus perpetuating chronic inflammation. Conversely, high dopamine concentrations in the gut of healthy individuals would stimulate DAR2, promoting the production of the anti-inflammatory cytokine IL-10 by CD4^+^ T cells (42) and suppressing both increased motility and ulcer development (102) (Table 1).

Taken together, the data discussed here support the hypothesis that dopamine has an overall protective role in the intestinal environment by acting over muscles and promoting the relaxation of the gut and preventing ulcer and lesion development, which can predispose individuals to develop intestinal inflammation. This protective role is most probably lost during colitis onset, since several lines of evidence indicate that dopamine levels decrease upon intestinal inflammation; and under these conditions, low dopamine levels may stimulate both the innate and adaptive compartments to produce highly inflammatory cytokines, favoring the development of colitis. Exploration of this hypothesis could lead to the development of novel therapies directed to dopaminergic targets that may be useful in the control of intestinal inflammation in IBD patients.

## Dopamine and Synovial Inflammation in Rheumatoid Arthritis

The etiology of RA remains under investigation, but accumulating evidence suggests that the disease develops in genetically predisposed individuals after exposure to environmental triggers. In addition to the presence of autoantibodies such as rheumatoid factor and cytokines, there is an impaired expression of several B and T lymphocyte subsets. Initial indications that dopamine signaling pathways are involved in the pathogenesis of RA come from the negative association between schizophrenia, thought to be dependent on excessive response of DAR2, and RA. Thus, in patients with schizophrenia, the median incidence rate of RA approximates 0.09%, a rate that is only one-tenth of that seen in RA in the general population (106). This negative association may suggest that excessive stimulation of DAR2-like receptors by dopamine may prevent the onset of RA.

In *ex vivo* studies, DAR1 was expressed at high levels in human naive CD4^+^ T cells, and dopamine could increase the cAMP concentrations in T cells via DAR1-like receptors, and subsequently induce the secretion of IL-4 and IL-5 (107). Additionally, dopamine increases IL-6-dependent IL-17 secretion from human T cells. Importantly, DAR1-like receptor antagonists inhibit dopamine-mediated IL-6 and IL-17 secretion from human T cells. This inhibitory effect by antagonizing DAR1-like receptors, which was demonstrated with SCH-23390 and LE300 DAR1-like receptor antagonists, involves the dopamine-mediated IL-6–Th17 axis (107).

Because dopamine is detectable in the inflamed synovial tissue of RA patients (107), it was of interest to investigate the effects of DAR-like receptor antagonists on RA development in animal models. DBA/1 mice immunized with type II collagen develop CIA, and treatment of the arthritic mice with the selective DAR1 antagonist SCH-23390 suppressed CIA severity (108). However, the treatment did not affect serum levels of antibodies to type II collagen or the Th1/Th17 differentiation of splenic T cells in the treated animals. When bone marrow-derived macrophages were stimulated *in vitro* in the presence of the DAR1 antagonist SCH23390, alteration of inflammatory cytokine expression was not observed, but their *in vitro* differentiation to osteoclasts was inhibited. Importantly, co-administration of the selective DAR1 agonist A68930 abrogated the *in vivo* anti-arthritic effect and the *in vitro* suppression of osteoclastogenesis by the DAR1 antagonist. The results suggest that DAR1 blockade could represent a potentially novel approach for RA treatment. Its effect could be partly attributable to the inhibition of osteoclastogenesis. DCs could synthesize and store dopamine, then release it to naive CD4^+^ T cells upon DC–T cell interactions, and, thereby, affect helper T cell differentiation. Such model is plausible because DCs have been proposed to play a pivotal role in the initiation and perpetuation of RA by presentation of arthritogenic antigens to T cells.

The effect of DAR-like receptor antagonists was also tested in rheumatoid synovitis of SCID mice engrafted with human RA synovium (107). Macroscopically, remarkable retraction of synovial tissue was observed in mice that received a DAR2-like receptor antagonist. In contrast, vascular proliferation and tissue enlargement were observed in mice that received the DAR2-like receptor antagonist. In the RA synovial/SCID mouse chimera model, although the selective DAR2-like receptor antagonist haloperidol significantly induced accumulation of IL-6^+^ and IL-17^+^ T cells, and exacerbated cartilage destruction, SCH-23390 strongly suppressed these responses. Taken together, the findings suggest that dopamine released by DCs acts on the IL-6–Th17 axis and causes aggravation of RA synovial inflammation.

During the last decade, the SNS has been proposed to be involved in the pathogenesis of RA. In experimental studies, CIA developed with less severity during early manifestations of disease in sympathectomized mice than in animals bearing intact SNS (27), suggesting a pro-inflammatory role exerted by SNS. *In vivo*, adoptive transfer of Tregs showed that an intact SNS attenuates suppressive functions of Tregs during CIA, and results in a significant increase of disease severity (27). In addition, recent studies revealed that sympathetic nerve fibers are lost in inflamed synovial tissue and, importantly, dopamine and NE have been described to induce repulsion of sympathetic nerve fibers (109). Of note, dopamine and NE are actively produced by synovial TH^+^ leukocytes during RA, independently of SNS function (110). Importantly, TH^+^ cells have been found in synovial tissue of RA patients, but not in healthy controls. Moreover, those TH^+^ cells start to replace sympathetic nerve fibers around the onset of disease, and modulation of locally produced catecholamines has strong anti-inflammatory effects *in vivo* in CIA and *in vitro* in samples obtained from synovial tissues of RA patients (110). More recently, *in vitro* experiments have shown that hypoxia stimulates expression of TH in synovial cells with a consequent synthesis of catecholamines and inhibition of TNF-α production in samples obtained from RA patients (111). Furthermore, the therapeutic relevance of these findings has been probed in a mouse model wherein adoptive transfer of TH^+^ neuronal cells generated from mouse mesenchymal stem cells into arthritic mice decreased disease severity (111). When TH^+^ neuronal cells were previously depleted of catecholamines by treatment with 6-hydroxydopamine, the therapeutic effect in CIA was lost (111). Thus, these data indicate that dopamine and other catecholamines produced by leukocytes play an important regulatory role in the physiopathology of RA.

In human naive CD4^+^ T cells, dopamine increases IL-6-dependent IL-17 production via DAR-like receptors, in response to anti-CD3 plus anti-CD28 antibodies. Furthermore, dopamine localizes with DCs in the synovial tissue of RA patients and is significantly increased in RA synovial fluid (107). Such observations, together with the preclinical studies of dopamine receptor antagonists prompted studies in patients. This resulted in several clinical trials that evaluated the effect of bromocriptine – a dopaminergic agonist that exerts a therapeutic activity through action on peripheral DARs – on RA disease activity. Clinical therapeutic trials using 2.5–30 mg of bromocriptine per day in a single or divided dose have shown efficacy with minimal side effects in the treatment of rheumatic diseases (112). Bromocriptine administration induced immunosuppression of several immune parameters and was associated with improvements in morning stiffness, grip strength, numbers of swollen/painful joints, and the Health Assessment Questionnaire disability index. Even though double-blind, placebo-controlled studies are limited, the clinical observations and trials support the use of bromocriptine, which is relatively safe, as a non-standard primary or adjunctive therapy in the treatment of recalcitrant RA, and associated conditions unresponsive to traditional approaches.

Cabergoline is another potent dopamine receptor agonist of DAR2 receptors. It exhibits a higher affinity for DAR2s; it also has less severe side effects and more convenient dosing schedule than bromocriptine. Its administration once or twice a week has much less tendency to cause nausea than bromocriptine. A pilot randomized double-blind clinical trial carried out in 10 patients with active RA reported improvement of tender and swollen joints (113). In addition, patient assessment of pain and global assessment of disease activity were significant when patients were treated with the long-acting cabergoline, a drug whose potential side-effects and cost are acceptable (Table 1).

## Lupus Neuropsychiatric Dysfunctions and the Dopaminergic System

Systemic lupus erythematosus is an autoimmune disease characterized by the involvement of multiple organs, including the kidneys and the brain. The disease course is variable and unpredictable, and often difficult to treat. It is characterized by immune deregulation and production of autoantibodies directed toward a variety of nuclear antigens, such as chromatin, RNP, Ro, La, Sm, Ku, and DNA. Despite considerable interest, the primary disturbances that lead to the production of pathogenic autoantibodies in SLE remain to be elucidated. It is, however, clear that the disease is multifactorial, with genetic and environmental factors contributing to its pathogenesis (114, 115). Several observations suggest that hormones are crucial regulators of SLE activity, including its preponderance in women, the fluctuations of disease activity with the menstrual cycle, the tendency of the disease to flare during pregnancy, and the remissions after menopause or cyclophosphomide-induced ovarian failure (116).

Neurologic manifestations of unknown etiology are common in SLE and have been proposed to represent a more severe form of the disease, occurring in up to 75% of patients (117). They range from diffuse CNS disorders, i.e., acute confusional state, psychosis, anxiety, and depressive disorders, clinical to subclinical cognitive disorders of variable functional significance, to CNS syndromes, i.e., seizures, cerebrovascular disease, chorea and myelopathy, transverse myelitis, demyelinating syndrome and aseptic meningitis, headaches, and PNS disorders, i.e., polyneuropathies and mononeuropathies, autonomic disorders, plexopathy, and myasthenia gravis. The neurologic manifestations may also include Parkinsonian-like deficits and changes in the basal ganglia. Although a pathologic neuro-immuno-endocrine circuitry has not yet been elucidated, significant loss of central neurons seems to underlie changes in sensorimotor function and behavior in many SLE patients (117). Support for the notion that behavioral impairments and neuronal demise are a consequence of autoimmunity comes from several case studies in which cyclophosphamide effectively reversed Parkinsonian-manifestations in SLE patients.

It is possible that disruption of the BBB and anti-neuronal autoantibodies account for CNS manifestations of the disease. Indeed, antibodies reacting with neuronal cell lines and brain tissue have been reported in the sera and CSF of patients with CNS-lupus, but they are also found in lupus patients with no clinical evidence of CNS involvement. Yet, specific autoantibodies may account for certain behavioral impairments (118). For example, a single injection of anti-dopamine antibodies reduces motor activity in healthy mice. In parallel, autoantibodies targeting dopaminergic neurons were associated with rapidly progressing Parkinsonian symptomatology in a SLE patient (119). Consistently, deposition of antigen–antibody complexes in choroidal blood vessels has been associated with neuropsychiatric dysfunction.

Systemic lupus erythematosus is also frequently accompanied by psychiatric manifestations of unknown origin. Although damage of central neurons had been documented, little is known about the neurotransmitter systems affected by the autoimmune/inflammatory process. Studies in lupus-prone MRL-lpr mice point to imbalanced dopamine function and neurodegeneration in dopamine-rich brain regions (120). Reminiscent of symptoms seen in patients, diseased MRL-lpr mice have impaired coordination in a beam-walking task and neurological deficits, and the accelerated autoimmune manifestations coincide with a deviation in their behavioral performance from congenic controls.

The series of performance deficits in motivated behavior, emotional reactivity, and learning/memory capacity in MRL-lpr mice have been operationally defined as “autoimmunity-associated behavioral syndrome” or AABS (121), and their physiopathology has been the focus of investigation. Significant changes in brain morphology have been observed at the onset of lupus-like disease in the MRL-lpr substrain, and post-mortem analysis revealed impaired catabolism of neurotransmitters in brains of lupic mice. Interestingly, autoimmune MRL-lpr mice exhibit behaviors reminiscent of stressed animals in a number of performance tests. The fact that the dopaminergic system of the mesencephalon is important in the control of movements, emotion, and motivated behavior, raises the issue of whether autoimmunity and inflammation are associated with midbrain degeneration, and whether anhedonic- and depressive-like behaviors may reflect an outcome of autoimmunity-induced damage on the mesolimbic dopaminergic system.

MRL-lpr brains show increased dopamine levels in the paraventricular nucleus (PVN) and median eminence, decreased concentrations of serotonin in the PVN, enhanced levels of serotonin in the hippocampus, and decreased NE levels in the prefrontal cortex (121). Importantly, the behavioral deficits correlate with changes in PVN and median eminence, suggesting that an imbalanced neurotransmitter regulation of the hypothalamus–pituitary axis plays a role in the etiology of behavioral dysfunctions induced by the autoimmune disease in MRL-lpr mice.

The causative role of autoimmunity and inflammation in the pathogenesis of AABS is supported by studies using the immunosuppressive drug cyclophosphamide, which prevents some behavioral deficits in lupus animals (122). More specifically, this drug prevents anxiety- and depressive-like behaviors as indicated by the restoration of novel object exploration, increased responsiveness to a sweet palatable solution, and reduced floating in the forced swimming test (FST).

Deficits in neurotransmitter catabolism are known to often be a consequence of aberrant synthesis and/or enzymatic activity, and excessive levels of dopamine can be neurotoxic. Accumulation of dopamine catabolism metabolites could contribute to the reduced density of dopaminergic cells. Regarding experimental lupus, elevated levels of dopamine in the brains of MRL-lpr mice and their increased sensitivity to the dopamine receptor agonist quinpirole are observations consistent with this notion (122).

There is now evidence that damage to central dopaminergic circuits in MRL-lpr brains accounts for some behavioral deficits. For example, chronic injection with the selective DAR2/DAR3 agonist quinpirole induced self-injurious behavior in lupus mice (121). Similarly, rotational behavior increased in MRL-lpr mice following acute injection with the selective DAR2/DAR3 dopamine agonist apomorphine. In the sucrose preference test, acute injection with the indirect dopamine agonist d-amphetamine failed to alter the response rates of diseased animals to sucrose solutions (120), while chronic treatment increased their mobility in the FST. Immunosuppressive treatment, suppressing autoimmunity and preventing hippocampal damage, circumvented an age-related decline in spatial memory and retrieval. Taken together, these results link neuropathological findings of dopaminergic cell death in nigrostriatal, mesolimbic, and mesocortical pathways to certain behavioral deficits (e.g., locomotor, motivated, and learning behaviors) in lupus-prone animals. Although the contribution of peripheral disease manifestations to behavioral performance cannot be excluded, these pharmacological results indicate that anhedonic- and depressive-like behaviors are a consequence of disease-driven damage to several dopamine systems in MRL-lpr brains.

As discussed above, human lymphocytes express all subtypes of DARs (DAR1–DAR5), each of which exerts specific actions on the regulation of lymphocyte functions. Since there are indications that neuromediators are involved in SLE pathogenesis, it was important to assess expression of dopamine receptor genes in this disease. A study of a cohort of SLE patients by sybergreen-based real-time PCR revealed that all receptors are expressed in lupus PBMCs. In addition, DAR2 was underexpressed, and DAR4 was overexpressed, as compared to control individuals (123). Cell sorting experiments of peripheral T and B lymphocytes disclosed that the altered DAR2 and DAR4 expressions were borne by T cells. These data support the view that these distorted expressions of DAR2 and DAR4 play a role in the pathophysiology of SLE. This assumption converges with the observation that lupus-prone MRL-*lpr* mice exhibit increased dopamine levels in specific regions of the brain, e.g., the PVN and median eminence (121).

It should be emphasized that changes in mRNA levels are often not as pronounced as the resulting differences in protein amounts. Therefore, comparison of the expression of DARs at the protein level could reveal additional distortions in SLE patients and in other lymphocytes subsets. A multicolor flow cytometric analysis of these receptors with highly specific antibodies or fluorochrome-labeled ligands and additional staining for different leukocyte populations will represent a fast, accurate, and easy method to assess DAR expression in blood samples. Such an approach would allow easy screening of a high number of patients and testing if the expression levels of DAR2 and DAR4 on T cells can be used as a diagnostic marker.

The observed changes in DAR expression in lymphocytes could influence immune functions in SLE patients through several mechanisms. First, stimulation of DAR2 in T cells has been shown to have functional consequences. Thus, DAR2 was found to be effective in regulating the activation and differentiation of naive CD4^+^ T cells by promoting polarization toward regulatory Treg cells (4, 42, 44, 47). In SLE patients, Treg cell function and/or numbers were reportedly diminished, which is consistent with the observation that expression of the Treg cell-promoting DAR2 is decreased in lupus (123). Second, DR4 is expressed in unstimulated human T cells, and is effective in regulating the activation and differentiation of naive CD4^+^ cells by triggering T cell quiescence (4, 42, 44, 47). Its activation is associated with the expression of KLF2, a transcription factor that regulates T cell quiescence (124) and permits a functional link between the nervous system and T cells. In SLE, there is an uncontrolled T cell proliferation that is thought to play an important pathogenic role. Together with the overexpression of DAR4 on lupus T cells (123), the observations (44) suggest that inducing quiescence using specific DAR4 agonists may represent a useful strategy in the treatment of this disease.

It has been hypothesized that the dopamine analog bromocriptine has the potential to suppress autoimmune disease. This rationale was applied to the treatment of SLE and in its (NZB × NZW) F_1_ mouse model. Treatment with bromocriptine was effective in treating the autoimmune disease in this experimental model. When treatment was started before the appearance of clinical disease, bromocriptine could slow the course of SLE in (NZB × NZW) F_1_ mice. In addition, this drug was effective in treating established disease in this model (125). In another animal model of SLE, simultaneous treatment with estrogen and the dopaminergic agent bromocriptine prevented the development of a lupus-like syndrome in BALB/c mice that express a transgene encoding a pathogenic anti-dsDNA antibody (126).

In clinical trials, bromocriptine showed evidence that it had a therapeutic effect in treating human lupus. For example, treatment with bromocriptine was found to be beneficial in SLE patients with mild to moderately active disease, leading to decreased serum immunoglobulin and anti-DNA antibody levels (112). Discontinuation of bromocriptine was followed by a flare of disease activity. In another clinical trial, the therapeutic effect of bromocriptine was found to be comparable to that of hydroxychloroquine, a well-accepted treatment for cutaneous and articular manifestations of SLE (125). The fact that bromocriptine was effective in treating (NZB × NZW) F_1_ mice, the beneficial therapeutic effects in human trials, and the low toxicity of the drug represent a solid rationale for undertaking further therapeutic trials. Thus, these observations suggest that alterations in dopamine and/or its receptors could be associated with the physiopathology of SLE (Table 1).

## Conclusions

Accumulating pharmacological and genetic evidence has indicated a crucial role of dopamine in the regulation of inflammatory responses involved in autoimmune disorders. Altered levels of dopamine in inflamed tissues and deregulated expression of the dopaminergic machinery in immune cells have been consistently associated with the physiopathology of autoimmune diseases in patients and in animal models, including MS, IBDs, SLE, and RA. Accordingly, drugs targeting the dopaminergic machinery have proven to be anti-inflammatory *in vitro* and *in vivo* in animal models and, in some cases, also in human patients. Understanding the precise mechanisms involving dopaminergic-mediated regulation of the immune system and their alterations in autoimmune disorders would allow development of drugs that could have beneficial effects for the treatment of autoimmunity. Despite current efforts in this field focusing in pharmacologically targeting components of dopaminergic machinery as a therapeutic approach *in vivo* in animal models of autoimmune diseases, there is a lack of available specific drugs, i.e., targeting DAR1 and DAR5. Therefore, future efforts should focus on evaluation of the targeting potential of these dopaminergic components in a receptor-specific manner (for example, by developing new agonists/antagonists specific for a particular receptor, or, alternatively, targeting specific receptor expression, i.e., by inducing ectopic up- or down-regulation with viral vectors). Furthermore, the complexity of dopaminergic regulation of the immune response, which involves several different DARs expressed in diverse leukocyte types, suggests that future therapies should point toward the development of strategies to target specific DARs or enzymes in precise cell populations. In this regard, recent studies have shown that adoptive transfer of cells with an altered dopaminergic machinery may decrease disease severity in some animal models of autoimmunity. Finally, not only targeting of neurotransmitter receptors should be assessed in isolation as therapeutic approaches in immune-related diseases, but also targeting different receptors in combination should be considered.

## Conflict of Interest Statement

The authors declare that the research was conducted in the absence of any commercial or financial relationships that could be construed as a potential conflict of interest.
